# Physical Activity Is Associated with Gut Microbiome Features and Organic Acid Patterns in Adults Consuming Plant-Rich Diets: An Exploratory Cross-Sectional Study

**DOI:** 10.3390/biology15060507

**Published:** 2026-03-21

**Authors:** Ramona Alina Tomuța, Alexandra Caltea, Marc Cristian Ghitea, Evelin Claudia Ghitea, Maria Flavia Gîtea, Timea Claudia Ghitea, Florin Banica

**Affiliations:** 1 Doctoral School of Biological and Biomedical Sciences, University of Oradea, 1 University Street, 410087 Oradea, Romania; yasmine_tomas@yahoo.com; 2 Surgery Department, Faculty of Medicine and Pharmacy, University of Oradea, 1 University Street, 410087 Oradea, Romania; calteaalexandra@gmail.com; 3 Faculty of Medicine and Pharmacy, University of Oradea, 410068 Oradea, Romania; ghitea.marccristian@student.uoradea.ro (M.C.G.); ghitea.evelinclaudia@student.uoradea.ro (E.C.G.); gitea.mariaflavia@student.uoradea.ro (M.F.G.); 4 Pharmacy Department, Faculty of Medicine and Pharmacy, University of Oradea, 1 University Street, 410087 Oradea, Romania; florinbanica1@gmail.com

**Keywords:** physical activity, plant-based diet, gut microbiome, organic acids, microbiome-related metabolites, gastrointestinal symptoms, metabolic resilience, exploratory study

## Abstract

Background: Plant-rich dietary patterns are widely associated with metabolic and gastrointestinal health benefits. However, individuals consuming predominantly plant-based foods may also experience chronic low-dose exposure to dietary pesticide residues. At the same time, physical activity is recognized as an important lifestyle factor influencing metabolic health and gut microbiome composition. How microbiome features and microbiome-related metabolic profiles vary according to physical activity level in adults consuming plant-rich diets and reporting gastrointestinal symptoms remains insufficiently characterized. Objective: To explore associations between physical activity level, gut microbiome characteristics, and urinary organic acid patterns in adults consuming predominantly plant-rich diets and experiencing gastrointestinal symptoms, within a cohort characterized by comparable estimated dietary pesticide exposure used as a contextual dietary background variable. Methods: This cross-sectional observational study included 93 adults consuming ≥50% plant-based foods for at least six months and reporting persistent gastrointestinal symptoms. Participants were stratified according to physical activity level using WHO-based thresholds (<150 vs. ≥150 min/week of moderate-intensity activity). Stool microbiota were assessed using a targeted quantitative PCR panel, and microbial diversity was summarized using a laboratory-derived Shannon index. A voluntary subgroup (*n* = 50) underwent targeted urinary organic acid analysis (LC–MS/MS). Dietary pesticide exposure was indirectly estimated using national surveillance data combined with individual dietary records and was applied uniformly across groups. Analyses were primarily descriptive and exploratory; results are presented as associations. Results: Estimated dietary pesticide exposure did not differ between physical activity groups. Participants with lower physical activity were older and exhibited lower microbial diversity and a higher prevalence of reduced abundance in selected commensal taxa. Differences were observed in selected intermediary organic acid markers, while no statistically significant difference was found for the bile acid-related indicator. Several cross-domain correlations were identified between microbial features and metabolite patterns. However, given the cross-sectional design, age imbalance between groups, and subgroup-based metabolomic analyses, the findings should be interpreted as hypothesis-generating rather than indicative of independent effects of physical activity. Conclusions: In adults consuming plant-rich diets and reporting gastrointestinal symptoms, physical activity level was associated with distinct microbiome and microbiome-related metabolic patterns under comparable estimated dietary pesticide exposure. These findings highlight the potential contribution of lifestyle factors to interindividual variability in gut microbial and metabolic profiles, while underscoring the need for age-adjusted, longitudinal, and biomarker-based studies to clarify directionality and mechanisms.

## Simple Summary

People who eat mostly fruits, vegetables, and other plant-based foods are often considered to have a healthy diet. However, these foods can also contain small amounts of pesticide residues, especially when they are conventionally grown. At the same time, lifestyle factors such as physical activity may influence how the body responds to dietary and environmental exposures. In this study, we examined adults who followed predominantly plant-based diets and reported digestive symptoms. All participants were exposed to similar levels of pesticide residues through their daily food intake. We compared individuals with lower and higher levels of physical activity to see whether lifestyle differences were associated with changes in gut bacteria and metabolic markers. We found that people with lower physical activity more often had reduced diversity of gut bacteria and different patterns of bacteria linked to digestion and metabolism. They showed differences in selected intermediary metabolic markers, while several other metabolic pathways did not differ between groups. In contrast, individuals who were more physically active tended to show more balanced gut bacterial and metabolic profiles, despite having similar dietary exposure. These findings suggest that physical activity may play an important role in shaping gut health and metabolic responses in people consuming plant-rich diets. Understanding how lifestyle factors interact with diet may help improve strategies for maintaining digestive and metabolic well-being.

## 1. Introduction

Plant-rich dietary patterns are widely promoted for their benefits for cardiometabolic health, gastrointestinal function, and environmental sustainability. Diets characterized by high intake of fruits, vegetables, legumes, and whole grains provide dietary fiber, polyphenols, and bioactive compounds known to influence gut microbial composition and metabolic pathways. At the same time, individuals consuming predominantly plant-based foods may be chronically exposed to low-dose mixtures of pesticide residues present in conventionally grown produce. In the present study, pesticide exposure was not directly measured through biomonitoring, but indirectly estimated using national food surveillance data combined with individual dietary records. Although regulatory assessments evaluate pesticide safety at the compound level, real-world dietary exposure typically involves complex residue mixtures. In the present study, such exposure was considered a shared environmental background condition rather than a primary explanatory variable [1,2,3,4]. Although pesticide residues are frequently discussed in relation to plant-based diets, the present study did not aim to test the mechanistic effects of pesticide exposure on the microbiome. Instead, estimated dietary pesticide exposure was considered a contextual environmental background shared by participants consuming similar plant-rich diets. The analytical focus of the study was the association between physical activity level and microbiome-related biological patterns within this dietary context.

The gastrointestinal tract represents a dynamic interface between diet, host physiology, and microbial ecology. The gut microbiome plays a central role in nutrient metabolism, bile acid transformation, short-chain fatty acid production, immune signaling, and maintenance of intestinal barrier function [5,6]. Alterations in microbial diversity and taxonomic composition have been associated with gastrointestinal symptoms, low-grade inflammation, and metabolic dysregulation. In individuals reporting functional gastrointestinal complaints, microbiome-related metabolic patterns may be particularly relevant for understanding symptom variability and host–microbe interactions [7].

Beyond microbial composition alone, microbiome-derived and microbiome-associated metabolites provide functional insight into host–microbiome dynamics [8,9]. Urinary organic acids, including intermediates of energy metabolism and microbial-derived compounds such as hippurate and dicarboxylic acids, are increasingly used in exploratory clinical settings to characterize metabolic phenotypes [10,11]. While such targeted panels do not provide comprehensive metabolomic coverage, they offer integrative information on intermediary metabolic pathways and potential microbiome-related metabolic outputs [12,13].

Physical activity is a well-established determinant of metabolic health and has emerged as an important factor associated with gut microbiome diversity and composition. Recent systematic reviews and meta-analyses have also reported associations between habitual physical activity and microbial diversity, as well as the abundance of short-chain fatty acid-producing taxa [14,15,16,17]. Observational and interventional studies suggest that regular physical activity is associated with higher microbial diversity, increased abundance of short-chain fatty acid-producing taxa, and improved metabolic flexibility. Conversely, lower physical activity levels have been linked to dysbiotic patterns and altered metabolic profiles. However, many previous studies have focused either on athletic populations or specific disease cohorts, and less is known about how physical activity relates to microbiome and metabolite patterns in adults consuming plant-rich diets and experiencing gastrointestinal symptoms [18,19]. Age is one of the strongest determinants of gut microbiome structure and metabolic physiology and is also closely linked to physical activity patterns in population-based cohorts. Consequently, interpretation of microbiome differences observed between activity groups requires careful consideration of potential age-related confounding. The present study therefore emphasizes descriptive associations rather than causal interpretations.

Many individuals adhering to plant-rich dietary patterns report gastrointestinal symptoms. These symptoms may arise from increased dietary fiber fermentation, higher intake of fermentable carbohydrates (FODMAPs), or coexistence of functional gastrointestinal conditions, which are commonly reported in individuals transitioning toward plant-dominant dietary patterns [20]. These symptoms are often considered functional in nature, yet they may coincide with measurable changes in microbiome-related metabolic profiles [21,22]. The extent to which lifestyle factors, particularly physical activity, shape microbiome characteristics and metabolite patterns in this context remains insufficiently explored [11,23,24,25]. The present cohort was intentionally restricted to individuals consuming predominantly plant-rich diets who also reported gastrointestinal symptoms, as these individuals frequently seek microbiome-related testing in clinical nutrition practice. Studying this population allows exploration of microbiome–metabolite patterns in a real-world context where dietary composition and digestive symptoms coexist. The ≥50% plant-based threshold was used pragmatically to capture individuals whose dietary intake was clearly dominated by plant foods while maintaining sufficient variability in dietary patterns.

Importantly, lifestyle factors rarely operate in isolation. In real-world settings, dietary patterns, environmental exposures, metabolic health status, and physical activity coexist and may collectively shape microbiome-related outcomes. Within this framework, examining associations between physical activity level, gut microbial features, and urinary organic acid profiles in a cohort sharing similar dietary characteristics may provide insight into interindividual variability in microbial–metabolic patterning.

Based on previous observations linking physical activity to gut microbial diversity and metabolic flexibility, we hypothesized that individuals reporting lower physical activity levels would exhibit less favorable microbiome diversity patterns and altered microbiome-associated metabolic markers compared with more physically active individuals, despite comparable dietary exposure conditions.

Therefore, the objective of this exploratory cross-sectional study was to investigate associations between physical activity level, gut microbiome characteristics, and targeted urinary organic acid patterns in adults consuming predominantly plant-rich diets and reporting gastrointestinal symptoms. Estimated dietary pesticide exposure was considered as a contextual background factor common to the cohort. Given the observational design, analyses were intended to identify associative patterns and generate hypotheses for future age-adjusted and longitudinal investigations.

## 2. Materials and Methods

### 2.1. Study Design and Participants

This exploratory cross-sectional observational study was conducted between January 2024 and May 2025 in Romania. The primary aim was to evaluate associations between physical activity level, gut microbiome features, and urinary organic acid patterns in adults consuming predominantly plant-rich diets and reporting gastrointestinal symptoms.

Given the observational design, analyses were intended to identify associative patterns rather than establish causality. The study protocol was approved by the Ethics Committee of the University of Oradea (approval code CEFMF/3, 30 October 2023), and all participants provided written informed consent.

### 2.2. Physical Activity Stratification

A total of 93 adults aged ≥18 years were included. Eligibility criteria were:

Inclusion criteria:

- Age ≥ 18 years
- Self-reported consumption of a predominantly plant-rich diet for ≥6 months
- Presence of persistent gastrointestinal symptoms (e.g., bloating, abdominal discomfort, altered bowel habits)
- No antibiotic use within the previous 3 months
- Willingness to provide stool samples

Exclusion criteria:

- Diagnosed gastrointestinal, hepatic, renal, or metabolic disease
- Pregnancy or breastfeeding
- Recent hospitalization (<6 months)
- Use of medications known to significantly alter gut microbiota

A predominantly plant-rich diet was operationally defined as ≥50% of total food intake derived from plant foods. This threshold was selected pragmatically to identify individuals whose diets were clearly dominated by plant-derived foods while maintaining variability typical of real-world dietary patterns. This threshold was used pragmatically for cohort characterization and was not intended to replace validated plant-based diet indices.

Participants were recruited consecutively from nutrition clinics and primary care offices.

### 2.3. Assessment of Physical Activity

Physical activity was assessed using the validated short-form International Physical Activity Questionnaire (IPAQ-SF). Because the questionnaire relies on self-reported activity levels, responses may be subject to recall bias or potential overestimation of physical activity. Participants were classified according to WHO recommendations [26]:

Low Physical Activity (LPA): <150 min/week of moderate-intensity activity.

Moderate-to-High Physical Activity (MHPA): ≥150 min/week of moderate or vigorous activity.

This stratification constituted the primary analytical grouping variable.

Because age differed substantially between physical activity groups, age was considered a potential confounder in subsequent analyses.

### 2.4. Gastrointestinal Symptom Assessment

Gastrointestinal symptoms were assessed using a structured questionnaire documenting the presence and duration of common symptoms (bloating, flatulence, diarrhea, constipation, abdominal pain).

A study-defined categorical symptom burden variable was created for exploratory correlation analyses:

1 = No/Minimal (≤2 symptoms, <6 months).

2 = Moderate (≥3 symptoms, <6 months).

3 = Higher burden (≥3 symptoms, ≥6 months).

This index was not a validated clinical score and was used exclusively for exploratory analyses.

### 2.5. Estimation of Dietary Pesticide Exposure

Dietary pesticide exposure was estimated using national surveillance data combined with individual dietary records. This approach represents population-level exposure modeling rather than individual biomonitoring.

Estimated daily intake for each participant was calculated by combining the following:

- Reported average daily intake (g/day) from 3-day dietary records
- Mean residue concentration values from national monitoring data
- Exposure metrics included:
- Estimated number of distinct residues per day
- Estimated cumulative daily intake (mg/day)

This estimation approach reflects population-level exposure modeling and does not represent individual biomonitoring. Because exposure estimation was applied uniformly across the cohort and did not differ between physical activity groups, pesticide exposure was treated as a shared background dietary condition rather than an explanatory variable.

Dietary pesticide exposure was estimated as a contextual environmental factor using national food surveillance bulletins issued by the Romanian Sanitary Veterinary and Food Safety Authority (ANSVSA) for the period 2024–2025 [27]. Reported residue concentrations (mg/kg) from raw, unwashed plant foods were combined with participants’ average portion sizes (g/day), obtained from three-day dietary records.

Estimated daily intake for each pesticide [28] was calculated using the formula below:

$$EDI=\sum_{i=1}^{n}(C_{i}\times IR_{i})\;$$

where:

- EDI = estimated daily intake (mg/day)
- C*_i_* = mean residue concentration of compound *i* (mg/kg)
- IR*_i_* = individual intake rate of food item *i* (kg/day)
- *n* = number of food items / exposure sources

### 2.6. Stool Microbiome Analysis

All participants provided stool samples, which were analyzed by GanzImmun Diagnostics (Mainz, Germany) [29]. Samples were stored at 2–8 °C and processed within 24 h of collection according to standardized laboratory protocols.

Microbial analysis was performed using a targeted quantitative PCR panel quantifying selected bacterial taxa commonly evaluated in clinical microbiome diagnostics. The panel included 25,000 bacterial taxa, allowing quantitative assessment of specific microbial groups relevant to gastrointestinal health. However, this approach does not provide the broader ecological resolution of sequencing-based microbiome profiling. Results were reported relative to laboratory-derived reference intervals.

This method does not provide comprehensive community-wide sequencing data (e.g., 16S rRNA sequencing or shotgun metagenomics). Therefore, diversity measures reflect the quantified taxa within the panel and should be interpreted accordingly. Microbial diversity was summarized using a Shannon diversity index calculated by the laboratory from the taxa included in the panel [30].

### 2.7. Urinary Organic Acid Analysis

A voluntary subgroup of 50 participants consented to additional metabolomic testing using a targeted urinary organic acid panel (Metabolomix+^®^, Genova Diagnostics, Asheville, NC, USA). The subgroup included 23 participants in the MHPA group and 27 participants in the LPA group [31].

Baseline demographic characteristics of participants included in the metabolomic subgroup were compared with those of the full cohort to assess potential selection bias. No major differences in sex distribution or BMI were observed; however, the voluntary nature of participation may still limit generalizability.

The panel includes selected intermediary metabolic markers, microbiome-related metabolites (e.g., hippurate), bile acid-related indicators, and markers associated with mitochondrial function.

The metabolomic subgroup was not randomly selected and may not be representative of the full cohort. Analyses of these data were considered exploratory.

Baseline characteristics of subgroup participants were compared with those of the full cohort to assess potential selection bias.

### 2.8. Statistical Analysis

Statistical analyses were performed using SPSS version 30 (IBM Corp., Armonk, NY, USA). Data normality was assessed using the Shapiro–Wilk test. Given the sample size and exploratory design, multivariable age-adjusted models were not performed. Therefore, residual confounding by age cannot be excluded.

Given the modest sample size and exploratory design, stable multivariable models including multiple covariates were considered statistically limited. However, exploratory regression analyses including age, sex, and BMI were conducted to evaluate potential confounding effects. Non-normally distributed continuous variables were analyzed using the Mann–Whitney U test or Kruskal–Wallis test, as appropriate. Categorical variables were compared using the chi-square test. Associations between variables were assessed using Spearman’s rank correlation coefficient. Correction for multiple comparisons was applied using the Benjamini–Hochberg false discovery rate procedure (FDR < 0.10).

Because age differed substantially between groups, sensitivity analyses were performed, including age as a covariate in exploratory partial correlations where sample size permitted.

Normality of bile acid-related metabolite indicator distribution was confirmed (Shapiro–Wilk *p* > 0.05); therefore, an independent *t*-test was applied. To partially address potential confounding, exploratory multivariable linear regression analyses were performed including age, sex, and BMI as covariates. Physical activity level was entered as the primary predictor variable. These models were considered exploratory due to the modest sample size.

**Figure 1:**
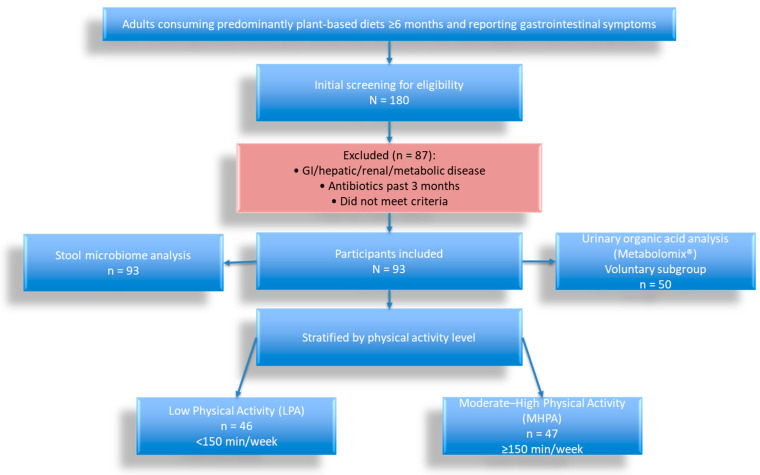
Flow Chart.

Primary analyses compared demographic characteristics, estimated dietary pesticide exposure, gut microbiome measures, and urinary organic acid profiles between the low and moderate-to-high physical activity groups. The study flowchart is presented in Figure 1.

All between-group comparisons should be interpreted as unadjusted unless explicitly stated otherwise.

## 3. Results

### 3.1. Participant Characteristics

A total of 93 adults consuming predominantly plant-based diets and reporting persistent gastrointestinal symptoms were included in the analysis. Participant age ranged from 29 to 87 years, and were stratified according to physical activity level into a moderate-to-high physical activity group (MHPA, *n* = 47) and a low physical activity group (LPA, *n* = 46). The most frequently reported gastrointestinal symptoms across the cohort included abdominal bloating, flatulence, and variable bowel habits. A substantial age difference between groups was observed. Participants in the low physical activity group were approximately 20 years older on average than those in the moderate-to-high activity group, a factor that represents a major potential confounder for microbiome and metabolic analyses (68.8 ± 6.6 years) compared with those in the MHPA group (49.3 ± 7.4 years).

Sex distribution was comparable between groups, with a slight predominance of male participants in both the MHPA and LPA groups (53.1% and 56.8%, respectively).

Anthropometric characteristics indicated a generally obese population in both groups. Body mass index was slightly higher in the MHPA group (35.23 ± 5.11 kg/m^2^) compared with the LPA group (33.55 ± 2.98 kg/m^2^), although this difference did not reach clinical relevance. Waist circumference values were similar between groups (111.39 ± 11.37 cm in MHPA vs. 108.55 ± 8.24 cm in LPA), which may reflect comparable levels of central adiposity.

These baseline characteristics indicate that while the two groups were similar with respect to sex distribution and adiposity measures, they differed substantially in age, a factor considered in the interpretation of subsequent microbiome and metabolomic findings (Table 1).

### 3.2. Estimated Dietary Pesticide Exposure

Estimated dietary pesticide exposure did not differ significantly between groups using either residue counts/day or cumulative intake (mg/day) (Table 2). Therefore, pesticide exposure was treated as a contextual background factor rather than a primary explanatory variable. On average, participants were estimated to be exposed to 12.4 ± 2.8 distinct pesticide residues per day, with individual values ranging from 9 to 17 different compounds.

Comparative analyses indicated no statistically significant differences in estimated dietary pesticide exposure between physical activity groups. The mean number of distinct residues consumed daily was 12.7 ± 2.6 in the low physical activity (LPA) group and 12.2 ± 3.0 in the moderate-to-high physical activity (MHPA) group (*p* = 0.41). Median values and exposure ranges were comparable between groups, and the proportion of individuals exposed to higher multi-residue counts (≥14 residues/day) did not differ significantly.

Estimated cumulative daily intake, expressed as total milligrams per day, was also similar between groups (0.23 ± 0.07 mg/day in LPA vs. 0.21 ± 0.08 mg/day in MHPA; *p* = 0.37). Across the cohort, cumulative daily intake values ranged from approximately 0.15 to 0.38 mg/day. The most frequently represented pesticide residues may reflect national monitoring trends and included compounds commonly detected in tomatoes, apples, cucumbers, onions, and potatoes, such as imidacloprid, difenoconazole, pyridaben, and chlorpyrifos.

**Figure 2:**
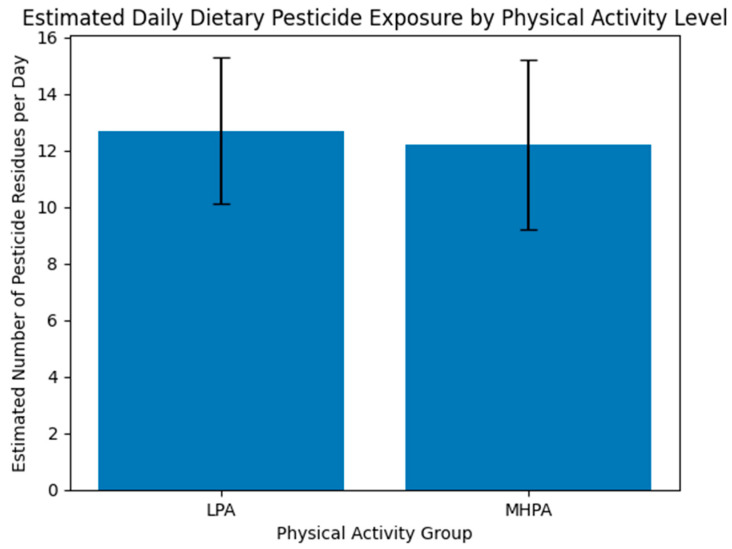
Estimated daily dietary pesticide exposure by physical activity group. Estimated number of distinct pesticide residues consumed per day in the low physical activity (LPA) and moderate-to-high physical activity (MHPA) groups. Values were derived from national food surveillance data combined with individual dietary records. No statistically significant differences were observed between groups.

Overall, these findings indicate that estimated dietary pesticide exposure was broadly comparable between participants with different physical activity levels. Consequently, any observed differences in gut microbiome characteristics or microbiome-related metabolic profiles described in subsequent analyses are unlikely to be explained solely by differences in estimated dietary pesticide intake (Table 2 and Figure 2).

### 3.3. Gut Microbiome Alterations

#### 3.3.1. Microbial Diversity

Gut microbial diversity differed between groups based on Shannon index values calculated from the targeted qPCR panel. Because diversity estimates were derived from a limited set of quantified taxa rather than full community sequencing, ecological interpretation should be made cautiously. Participants in the low physical activity (LPA) group exhibited lower microbial diversity compared with those in the moderate-to-high physical activity (MHPA) group, as reflected by Shannon diversity index values (median 2.71 vs. 3.06, *p* = 0.012).

When interpreted relative to laboratory reference thresholds, a reduced Shannon diversity index was observed in a higher proportion of individuals in the LPA group (72%) compared with the MHPA group (48%). In addition to lower central tendency values, diversity scores in the LPA group displayed greater dispersion, suggesting increased interindividual variability in gut microbial composition. In contrast, participants with higher levels of physical activity showed a more concentrated distribution of diversity values at higher levels.

Lower microbial diversity has been associated in previous studies with reduced functional redundancy and increased susceptibility to dysbiosis-related gastrointestinal complaints. In the present cohort, reduced diversity was more frequently observed among individuals with lower physical activity, despite comparable estimated dietary pesticide exposure across groups.

Overall, these findings indicate an association between physical activity level and gut microbial diversity in adults consuming predominantly plant-based diets and reporting gastrointestinal symptoms. Physical activity level was associated with variability in microbial diversity within this cohort; however, given the substantial age difference between groups, age-related microbiome variation may plausibly contribute to part of the observed signal.

#### 3.3.2. Beneficial Bacterial Taxa

A reduction in several beneficial commensal bacterial taxa was observed across the cohort, with a higher prevalence of below-reference values among participants in the low physical activity (LPA) group compared with those in the moderate-to-high physical activity (MHPA) group. The taxa most frequently affected included *Bifidobacterium adolescentis*, *Faecalibacterium prausnitzii*, and *Lactobacillus* spp., which are commonly regarded as indicators of a functionally balanced gut microbial ecosystem.

*Bifidobacterium adolescentis* levels below laboratory reference thresholds were observed in 89% of participants in the LPA group compared with 76% in the MHPA group. Similarly, *Faecalibacterium prausnitzii* was reduced in 85% of individuals with low physical activity versus 67% of those with higher activity levels. *Lactobacillus* spp. were below the detection threshold in most participants. However, this finding should be interpreted cautiously, as the targeted panel may underestimate the presence of certain taxa compared with sequencing-based microbiome approaches.

These findings indicate that reduced abundance of beneficial bacterial taxa was common in this symptomatic cohort, but occurred more frequently among individuals reporting lower levels of physical activity. Differences between groups were consistent with the diversity patterns described above, suggesting that physical activity level may be associated with interindividual variability in the presence of key commensal bacteria.

**Figure 3:**
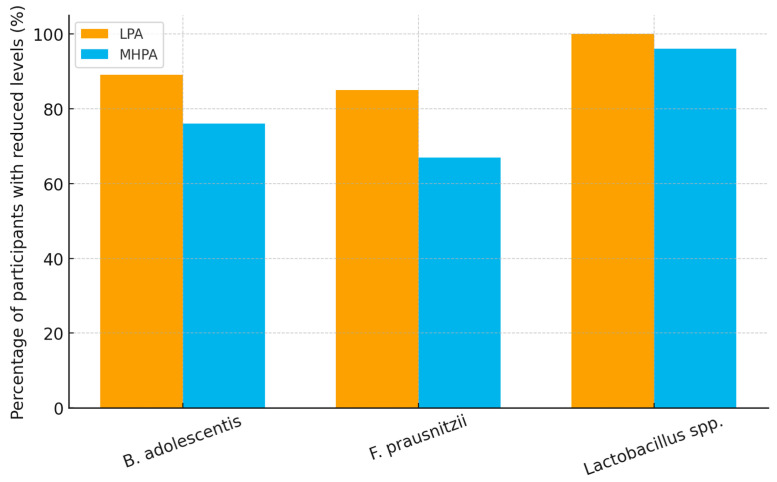
Prevalence of reduced beneficial bacterial taxa by physical activity level. Proportion of participants with below-reference levels of *Bifidobacterium adolescentis*, *Faecalibacterium prausnitzii*, and undetectable *Lactobacillus* spp. in the low physical activity (LPA) and moderate-to-high physical activity (MHPA) groups. A higher prevalence of reduced beneficial taxa was observed in participants with lower physical activity. Bifidobacterium reduced LPA: 41/46 (89%) and MHPA: 36/47 (76%).

Exploratory correlation analyses showed that lower relative abundance of *Faecalibacterium prausnitzii* was modestly associated with lower physical activity levels (Spearman’s ρ = −0.29, *p* = 0.021). An additional weak association was observed between *F. prausnitzii* abundance and estimated dietary pesticide exposure (ρ = −0.31, *p* = 0.014); however, given the comparable exposure levels between physical activity groups, these correlations should be interpreted cautiously and are reported for descriptive purposes only (Table 3 and Figure 3).

#### 3.3.3. Taxa Commonly Associated with Inflammatory Signaling

Differences were also observed between physical activity groups in bacterial taxa commonly described as pro-inflammatory or neuroactive. Participants in the low physical activity (LPA) group exhibited higher relative abundance of lipopolysaccharide (LPS)-associated taxa compared with those in the moderate-to-high physical activity (MHPA) group (median relative abundance 8.3% vs. 5.6%, *p* = 0.038).

In addition, taxa belonging to the genus *Oscillibacter*, which have been previously linked to valeric acid production and gut–brain signaling pathways, were above laboratory reference ranges in a higher proportion of LPA participants (72%) than MHPA participants (52%). Similarly, *Alistipes* spp. exceeded reference thresholds in 63% of individuals in the LPA group compared with 41% in the MHPA group.

**Figure 4:**
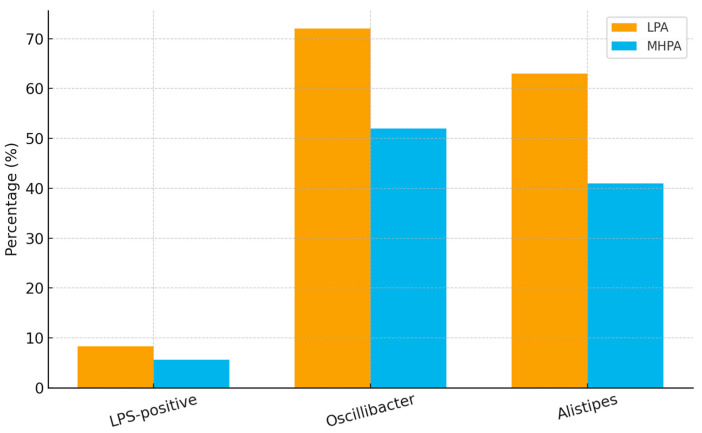
Prevalence of selected pro-inflammatory and neuroactive bacterial taxa by physical activity group. Relative abundance of LPS-associated taxa and proportion of participants with above-reference levels of *Oscillibacter* spp. and *Alistipes* spp. in the low physical activity (LPA) and moderate-to-high physical activity (MHPA) groups.

Lower physical activity was associated with a higher prevalence of taxa previously linked to inflammatory signaling. Importantly, these differences were observed in the context of comparable estimated dietary pesticide exposure between groups, suggesting that physical activity level may contribute to interindividual variability in gut microbial composition under similar dietary exposure conditions (Table 4 and Figure 4).

### 3.4. Urinary Organic Acid Profiles (Metabolomix+^®^ Subgroup)

The metabolomics subgroup included 50 participants (MHPA *n* = 23; LPA *n* = 27). All metabolomic analyses presented below refer exclusively to this subgroup.

All findings presented below are exploratory and should be interpreted in the context of subgroup design and cross-sectional analysis.

#### 3.4.1. Bile Acid-Related Indicators

The bile acid-related indicator (V22) did not differ significantly between physical activity groups (MHPA 6.11 ± 1.88 vs. LPA 6.60 ± 1.56; *p* = 0.188). The distributions showed substantial overlap, suggesting that bile acid-related metabolic variation does not represent a primary differentiating feature between groups in this cohort (Table 5).

#### 3.4.2. Intermediary/Microbial Metabolic Markers

Differences between physical activity groups were also observed in selected intermediary metabolic markers commonly interpreted as indicators of metabolic stress or altered intermediary metabolism.

Elevated values (above laboratory reference interval) were more frequent in LPA participants:

Pyroglutamate: 61% (LPA) vs. 29% (MHPA), *p* = 0.03.

Orotic acid: 44% vs. 18%, *p* = 0.047.

Dicarboxylic acids (adipate/suberate/ethylmalonate composite): 67% vs. 35%, *p* = 0.028.

These markers are often used in targeted organic acid panels to reflect glutathione turnover, urea cycle flux, and fatty acid oxidation patterns, and are commonly interpreted in targeted organic acid panels as indicators of intermediary metabolic variation; however, they should not be interpreted as validated clinical biomarkers of metabolic dysfunction.

However, given the subgroup design and age imbalance, exploratory age-adjusted analyses indicated attenuation of some associations, suggesting that age-related metabolic variation may partially contribute to observed differences.

No conclusions regarding metabolic dysfunction or mechanistic pathways can be drawn from these data (Table 6).

#### 3.4.3. Neuroactive and Indole-Derived Metabolites

Quantitative comparison of metabolites related to the following:

- Kynurenine pathway
- Catecholamine metabolism
- Indole-derived microbial metabolites did not reveal statistically significant differences between physical activity groups.

Distribution of values within laboratory reference intervals was comparable across groups, and the proportion of elevated results did not differ meaningfully.

These findings suggest that neuroactive and tryptophan-related metabolic pathways were not primary contributors to between-group variation in this cohort (Table 7).

Following quantitative comparison, neuroactive and indole-derived organic acids showed largely similar distributions between the two physical activity groups. Metabolites of the kynurenine pathway (kynurenic, quinolinic, and xanthurenic acids) were within laboratory reference ranges in most participants, with comparable proportions of elevated values in both MHPA and LPA groups. Likewise, catecholamine-related metabolites (homovanillic and vanilmandelic acids) and indole-derived compounds (5-hydroxyindoleacetic acid, indoleacetic acid, and phenylacetic acid) did not differ meaningfully between groups.

**Figure 5:**
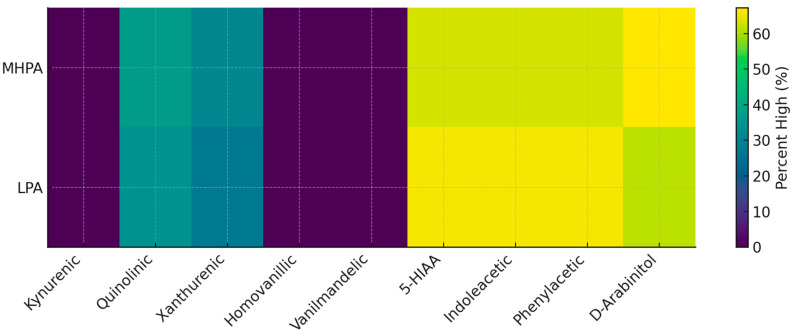
Distribution of neuroactive and indole-derived organic acids by physical activity group. Heatmap illustrating the proportion of participants with values above laboratory reference ranges for selected neuroactive and indole-derived organic acids in the moderate-to-high physical activity (MHPA) and low physical activity (LPA) groups. Comparable distributions across groups indicate that these pathways do not substantially differ by physical activity level in this cohort. The color scale represents the proportion of participants above reference values (%).

D-arabinitol, a marker often interpreted as reflecting yeast or fungal metabolic activity, was elevated in a substantial proportion of participants in both groups, with only minor differences in distribution. Overall, these findings suggest that neuroactive and indole-related metabolic pathways were not major contributors to the metabolic differences observed between physical activity groups in this cohort (Figure 5).

### 3.5. Integrative Correlation Analysis

Integrative correlation analyses were performed to explore associations among physical activity level, estimated dietary pesticide exposure, gut microbiome features, and selected organic acid metabolites. Given the cross-sectional design and subgroup analyses, these results are reported descriptively and interpreted as exploratory.

Lower physical activity levels were associated with less favorable microbiome and metabolomic patterns across multiple domains. In participants with low physical activity, reduced microbial diversity and higher relative abundance of LPS-associated taxa were more frequently observed. Although estimated dietary pesticide exposure did not differ between physical activity groups, weak to moderate correlations between exposure estimates and selected microbiome markers were more apparent in individuals with lower activity levels.

Several cross-domain associations were identified between gut microbial features and organic acid metabolites. Reduced abundance of *Faecalibacterium prausnitzii* was inversely correlated with urinary pyroglutamate concentrations (r = −0.42, *p* = 0.004), indicating a relationship between reduced abundance of butyrate-associated taxa and altered intermediary metabolic markers. Higher relative abundance of *Oscillibacter* spp. was positively correlated with valeric acid-related metabolites (r = 0.38, *p* = 0.009), consistent with previously reported metabolic characteristics of this genus.

Lower urinary hippurate levels were positively associated with reduced microbial diversity (r = 0.46, *p* = 0.003) and showed a weak inverse association with estimated dietary pesticide exposure (r = −0.33, *p* = 0.016). In addition, lower hippurate concentrations were associated with study-defined higher gastrointestinal symptom category, characterized by ≥3 concurrent symptoms persisting ≥6 months, suggesting a link between microbial metabolic output and symptom burden.

Associations were also observed between dysbiosis-related microbial features and bile acid-related metabolites. Participants exhibiting higher relative abundance of LPS-associated taxa and valeric acid-associated genera more frequently showed elevated unconjugated-to-conjugated bile acid ratios; however, these relationships were exploratory and did not imply directionality or causality.

**Figure 6:**
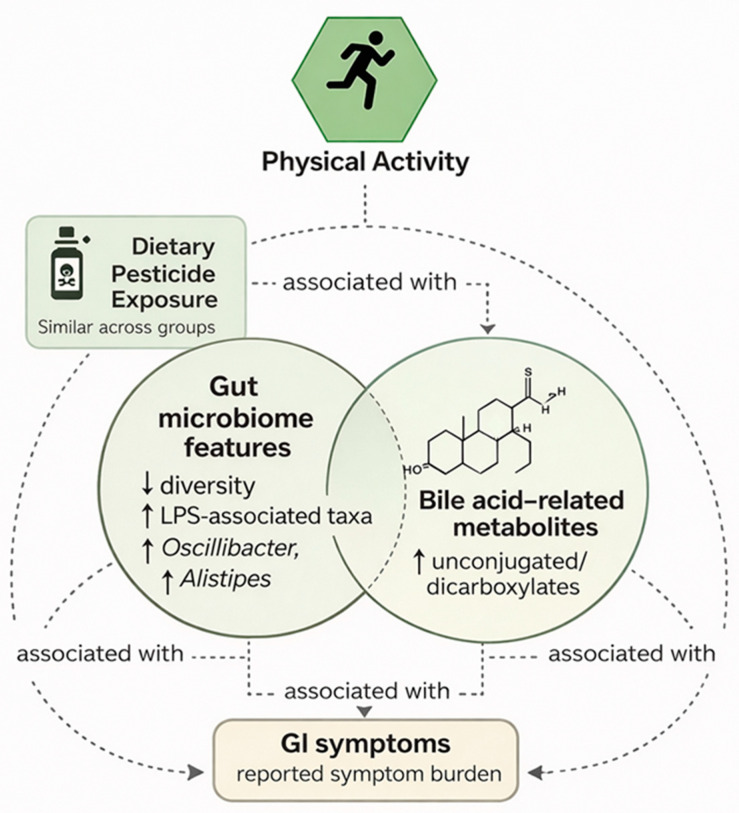
Conceptual representation of associations among dietary exposure, gut microbiome features, and bile acid-related metabolites. Schematic illustration depicting observed associations among estimated dietary pesticide exposure, gut microbiome characteristics, and bile acid-related metabolic markers. The diagram summarizes patterns identified in correlation analyses without implying causal directionality or mechanistic pathways. Arrows indicate the direction of change in biological parameters: ↓ indicates a decrease (e.g., reduced gut microbial diversity). ↑ indicates an increase (e.g., increased abundance of LPS-associated taxa, *Oscillibacter*, *Alistipes*, and increased levels of unconjugated or dicarboxylated bile acid metabolites).

Taken together, these correlation patterns suggest coordinated variation across physical activity level, gut microbial composition, and microbiome-related metabolic markers. These associations were observed in the context of comparable estimated dietary pesticide exposure across groups and highlight the complexity of host–microbiome–metabolite interactions in adults consuming plant-rich diets and reporting gastrointestinal symptoms (Table 8 and Figure 6).

Because these associations were derived from cross-sectional and partially subgroup-based data, they may reflect shared confounding factors (including age, lifestyle patterns, or metabolic status) rather than direct mechanistic coupling between microbial taxa and metabolites.

### 3.6. Multivariable Analysis

Exploratory multivariable regression analysis was performed to evaluate whether physical activity remained associated with the bile acid-related indicator after adjustment for age, sex, and BMI. In the adjusted model, physical activity was not independently associated with bile acid-related metabolite indicator (β = 0.27, *p* = 0.646). Age showed a borderline association (β = 0.042, *p* = 0.089), while sex remained a significant predictor (β = −1.19, *p* = 0.001). The overall model explained approximately 18% of the variance in bile acid-related metabolite indicator values (R^2^ = 0.18) (Table 9).

## 4. Discussion

### 4.1. Physical Activity and Gut Microbiome Features in a Plant-Rich Diet Context

In this exploratory cross-sectional study of adults consuming predominantly plant-rich diets and reporting gastrointestinal symptoms, physical activity level was associated with differences in gut microbiome characteristics. Specifically, participants with lower physical activity exhibited reduced Shannon diversity and a higher prevalence of below-reference values for selected commensal taxa, including *Faecalibacterium prausnitzii* and *Bifidobacterium adolescentis*. In addition, taxa commonly described in the literature as LPS-associated or potentially pro-inflammatory were more frequently elevated in the low physical activity group [32,33].

Importantly, estimated dietary pesticide exposure did not differ between physical activity groups, suggesting that the observed microbial differences are unlikely to be attributable to variations in modeled dietary residue intake within this cohort. In the present design, pesticide exposure functioned as a shared background condition rather than a discriminating variable [34].

The finding of lower microbial diversity among participants with lower physical activity is consistent with previous observational studies linking sedentary behavior to reduced microbial richness and altered taxonomic profiles. However, the magnitude of the age difference between groups (approximately 20 years) represents a major potential confounder. Age is a well-established determinant of gut microbiome composition, and part of the observed diversity differences may plausibly reflect age-related microbial shifts rather than physical activity per se. Therefore, the present findings should be interpreted as associations within a stratified cohort rather than as independent effects of physical activity [35,36,37].

### 4.2. Bile Acid-Related Indicators: Absence of Robust Between-Group Differences

In contrast to the initial exploratory hypothesis, analysis of the bile acid-related metabolite indicator did not reveal a statistically significant difference between physical activity groups (*p* = 0.188). Although mean values were numerically higher in the low physical activity group, substantial overlap between distributions was observed.

This lack of statistical separation suggests that bile acid-related metabolic variation does not represent a robust differentiating feature between physical activity groups in this cohort. While bile acids are increasingly recognized as mediators of gut–liver–microbiome interactions, the current data do not support a clear between-group divergence in this domain [38,39,40].

Given the subgroup design, cross-sectional framework, and age imbalance, bile acid-related findings should be considered descriptive rather than indicative of altered enterohepatic regulation or hepatic dysfunction [41].

### 4.3. Intermediary Metabolic Markers: Exploratory Differences with Age Influence

Selected intermediary metabolic markers, including pyroglutamate, orotic acid, and composite dicarboxylic acids, were more frequently elevated in participants with lower physical activity. These markers are commonly interpreted in targeted organic acid panels as reflecting glutathione turnover, urea cycle flux, and fatty acid oxidation patterns [42,43,44].

However, several important considerations apply:

- These analyses were performed in a voluntary subgroup.
- The study was not powered specifically for metabolomic comparisons.
- Age-adjusted exploratory analyses suggested attenuation of some associations.

Therefore, while these markers differed descriptively between groups, the findings should be interpreted cautiously. It remains plausible that age-related metabolic variation, rather than physical activity alone, contributes to these patterns.

No evidence from the present data supports conclusions regarding metabolic dysfunction, oxidative stress pathology, or mechanistic pathway activation.

Consequently, the metabolomic findings should primarily be interpreted as descriptive metabolic signatures within this cohort rather than as evidence of specific pathway activation.

### 4.4. Neuroactive and Indole-Derived Metabolites: No Evidence of Group Separation

Quantitative comparison of kynurenine pathway metabolites, catecholamine-related metabolites, indole-derived compounds, and D-arabinitol revealed no statistically significant differences between physical activity groups. Mean values and standard deviations demonstrated substantial overlap.

These findings indicate that neuroactive and tryptophan-related metabolic pathways do not meaningfully differentiate physical activity groups in this cohort. Consequently, earlier hypotheses regarding gut–brain axis divergence according to activity level are not supported by the present metabolomic data.

This negative result is important, as it refines the interpretation of microbiome-associated metabolic variation and prevents overextension of gut–brain mechanistic claims [45,46,47,48].

### 4.5. Integrative Associations: Microbial–Metabolic Correlation Patterns

Exploratory correlation analyses identified associations between selected microbial taxa and organic acid markers, including:

Inverse association between *Faecalibacterium prausnitzii* and pyroglutamate.

Positive association between *Oscillibacter* spp. and valeric acid-related metabolites.

Association between hippurate and microbial diversity.

These correlations suggest coordinated variation between microbial composition and selected metabolic outputs. However, correlation does not imply causation, and given multiple comparisons and subgroup analyses, these findings should be regarded as hypothesis-generating.

Notably, estimated dietary pesticide exposure showed only weak associations with selected microbial markers and did not differentiate physical activity groups, reinforcing the interpretation of pesticide exposure as contextual rather than explanatory within this study.

### 4.6. Interpretation Within the Context of Age Imbalance

A central limitation influencing interpretation is the substantial age difference between physical activity groups. Older participants were overrepresented in the low physical activity group, and aging is independently associated with the following:

- Reduced microbial diversity
- Altered taxonomic composition
- Changes in bile acid metabolism
- Altered intermediary metabolic markers

Because multivariable age-adjusted regression models were not performed, residual confounding by age cannot be excluded. Therefore, the observed associations between physical activity and microbiome/metabolomic features should be interpreted as stratified observational patterns rather than independent lifestyle effects.

Future studies should employ age-matched cohorts or multivariable models to disentangle age-related from activity-related variation.

### 4.7. Strengths, Limitations, and Future Directions

The study has several important limitations.

First, the cross-sectional design precludes causal inference.

Second, age differed substantially between physical activity groups and multivariable adjustment was limited.

Third, microbiome profiling relied on a targeted qPCR panel rather than sequencing-based methods, restricting ecological coverage.

Fourth, metabolomic analyses were performed in a voluntary subgroup.

Finally, pesticide exposure was estimated indirectly using surveillance data rather than individual biomonitoring.

Future research should employ longitudinal and interventional designs, incorporate direct measurements of pesticide biomarkers, and integrate multi-omics approaches to further elucidate how physical activity, diet, and environmental exposures interact to shape gut microbiome structure and metabolic outputs. Because age differed substantially between groups, residual confounding by age cannot be excluded despite cautious interpretation.

## 5. Conclusions

In this cross-sectional study of adults consuming predominantly plant-based diets and reporting gastrointestinal symptoms, physical activity level was associated with different patterns in gut microbiome composition and microbiome-related metabolic markers, despite comparable estimated dietary pesticide exposure across participants.

Individuals with lower physical activity more frequently exhibited reduced microbial diversity, lower prevalence of beneficial commensal taxa, and higher abundance of bacterial genera commonly reported in association with inflammatory or neuroactive metabolic pathways. No statistically significant differences were observed for the bile acid-related indicator, while selected intermediary organic acid markers showed exploratory between-group variation.

Importantly, estimated dietary pesticide exposure did not differ significantly between physical activity groups, indicating that dietary intake alone was unlikely to explain the observed biological variability.

Overall, the study identifies patterns of coordinated variation between physical activity level, microbiome features, and selected metabolic markers within a cohort consuming plant-rich diets and reporting gastrointestinal symptoms. However, due to the cross-sectional design, age imbalance between groups, and subgroup metabolomic analysis, the findings should be interpreted as exploratory observations rather than evidence of independent biological effects.

Future longitudinal and interventional studies incorporating direct exposure biomarkers and multi-omics approaches are warranted to further clarify how lifestyle factors, diet, and environmental exposures interact to shape gut microbial ecosystems and host metabolic phenotypes. These findings should be interpreted cautiously, given the cross-sectional design, age imbalance, and exploratory subgroup analyses.

## Figures and Tables

**Table 1:** Demographic and anthropometric characteristics of participants stratified by physical activity level.

Parameter	MHPA (*n* = 47)	LPA (*n* = 46)	*p*-Value *
Age (years), mean ± SD	49.34 ± 7.42	68.82 ± 6.55	<0.001
Body mass index (kg/m^2^), mean ± SD	35.23 ± 5.11	33.55 ± 2.98	0.09
Waist circumference (cm), mean ± SD	111.39 ± 11.37	108.55 ± 8.24	0.34

* Mann–Whitney U test; MHPA, moderate-to-high physical activity; LPA, low physical activity; SD, standard deviation.

**Table 2:** Estimated daily dietary pesticide exposure stratified by physical activity level.

Parameter	LPA (*n* = 46)	MHPA (*n* = 47)	*p*-Value
Number of pesticide residues/day, mean ± SD	12.7 ± 2.6	12.2 ± 3.0	0.41
Median (IQR)	13 (11–14)	12 (10–14)	—
Range (min–max)	9–17	9–17	—
Participants with ≥14 residues/day, *n* (%)	19 (38.8%)	13 (29.5%)	0.42
Participants with ≤10 residues/day, *n* (%)	9 (18.4%)	11 (25.0%)	0.58
Estimated cumulative intake (mg/day), mean ± SD	0.23 ± 0.07	0.21 ± 0.08	0.37

Estimated using national food surveillance data (ANSVSA 2024–2025) applied uniformly to individual dietary records.

**Table 3:** Prevalence of below-reference values for selected beneficial bacterial taxa stratified by physical activity level.

Bacterial Taxon	LPA (% Below Reference)	MHPA (% Below Reference)
*Bifidobacterium adolescentis*	89%	76%
*Faecalibacterium prausnitzii*	85%	67%
*Lactobacillus* spp.	100% undetectable	96% undetectable

**Table 4:** Prevalence of selected pro-inflammatory and neuroactive bacterial taxa stratified by physical activity level.

Taxon	LPA	MHPA
LPS-associated taxa (median relative abundance)	8.3%	5.6%
*Oscillibacter* spp. above reference range	72%	52%
*Alistipes* spp. above reference range	63%	41%

**Table 5:** Bile acid-related indicator by physical activity group.

Parameter	MHPA (*n* = 23)	LPA (*n* = 27)	*p*-Value
Bile Acid-Related Indicator (mean ± SD)	6.11 ± 1.88	6.60 ± 1.56	0.188

Test *t* independent: *p* = 0.188.

**Table 6:** Prevalence of elevated intermediary metabolic markers by physical activity group.

Marker	MHPA (*n* = 23) Elevated (%)	LPA (*n* = 27) Elevated (%)	*p*-Value
Pyroglutamate	29%	61%	0.03
Orotic Acid	18%	44%	0.047
Dicarboxylic Acids	35%	67%	0.028

**Table 7:** Neuroactive and indole-derived organic acids by physical activity group.

Metabolite	MHPA (*n* = 23) Mean ± SD	LPA (*n* = 27) Mean ± SD	*p*-Value
Kynurenic Acid	4.69 ± 1.05	4.58 ± 0.94	0.586
Quinolinic Acid	5.50 ± 3.41	5.48 ± 3.28	0.980
Xanthurenic Acid	0.74 ± 0.55	0.77 ± 0.55	0.827
Homovanillic Acid	2.85 ± 1.67	2.93 ± 1.70	0.826
Vanilmandelic Acid	1.24 ± 0.84	1.19 ± 0.90	0.760
5-HIAA	10.37 ± 0.67	10.26 ± 0.64	0.456
Indoleacetic Acid	3.73 ± 1.69	3.73 ± 1.75	0.987
Phenylacetic Acid	0.105 ± 0.044	0.098 ± 0.042	0.462
D-Arabinitol	24.20 ± 10.18	25.82 ± 9.83	0.448

**Table 8:** Exploratory correlations between physical activity, gut microbiome features, and organic acid metabolites.

Variable 1	Variable 2	r	*p*-Value
*Faecalibacterium prausnitzii*	Pyroglutamate	−0.42	0.004
*Oscillibacter* spp.	Valeric acid-related metabolites	0.38	0.009
Hippurate	Microbial diversity (Shannon index)	0.46	0.003
Hippurate	Estimated pesticide exposure	−0.33	0.016
Hippurate	Gastrointestinal symptom category (study-defined)	0.34	0.027

Reported *p*-values remained below the pre-specified FDR threshold (FDR < 0.10).

**Table 9:** Multivariable linear regression analysis evaluating the association between physical activity level and the bile acid-related metabolite indicator after adjustment for age, sex, and body mass index.

Predictor	Coefficient β	*p*-Value
Physical activity	0.27	0.646
Age	0.042	0.089
Sex	−1.19	0.001
BMI	0.029	0.489

Model global: R^2^ = 0.18, *p*(model) = 0.001.

## Data Availability

All processed data supporting the findings of this study are available in anonymized form through the Figshare repository (DOI: 10.6084/m9.figshare.30296359). Additional raw data can be made available by the corresponding author upon reasonable request, in compliance with national data protection regulations.
